# ‘God is the one who give child’: An abductive analysis of barriers to postnatal care using the Health Equity Implementation Framework

**DOI:** 10.21203/rs.3.rs-4102460/v1

**Published:** 2024-03-29

**Authors:** Emilie Egger, Befikadu Bitewulign, Humberto Gonzalez Rodriguez, Haley Case, Abiyou Kiflie Alemayehu, Elizabeth C. Rhodes, Abiy Seifu Estifanos, Kavita Singh, Dorka Woldesenbet Keraga, Marukh Zahid, Hema Magge, Dara Gleeson, Clare Barrington, Ashley Hagaman

**Affiliations:** Yale University School of Public Health; Institute for Healthcare Improvement; UNC Gillings School of Global Public Health: The University of North Carolina at Chapel Hill Gillings School of Global Public Health; CDC Foundation Inc: National Foundation for the Centers for Disease Control and Prevention Inc; Institute for Healthcare Improvement; Hubert Department of Global Health: Emory University Rollins School of Public Health; Addis Ababa University Department of Community Health: Addis Ababa University School of Public Health; The University of North Carolina at Chapel Hill Carolina Population Center; Addis Ababa University Department of Community Health: Addis Ababa University School of Public Health; Yale University School of Public Health; Addis Ababa University School of Public Health; Yale University School of Public Health; UNC Gillings School of Global Public Health: The University of North Carolina at Chapel Hill Gillings School of Global Public Health; Yale University School of Public Health

**Keywords:** maternal health, implementation, postnatal care, qualitative, abductive, Ethiopia

## Abstract

**Background:**

Postnatal care is recommended as a means of preventing maternal mortality during the postpartum period, but many women in low- and middle-income countries (LMICs) do not access care during this period. We set out to examine sociocultural preferences that have been portrayed as barriers to care.

**Methods:**

We performed an abductive analysis of 63 semi-structured interviews with women who had recently given birth in three regions of Ethiopia using the Health Equity Implementation Framework (HEIF) and an inductive-deductive codebook to understand why women in Ethiopia do not use recommended postnatal care.

**Results:**

We found that, in many cases, health providers do not consider women’s cultural safety a primary need, but rather as a barrier to care. However, women’s perceived refusal to participate in postnatal visits was, for many, an expression of agency and asserting their needs for cultural safety.

**Trial registration:**

n/a

**Conclusions:**

We propose adding cultural safety to HEIF as a process outcome, so that implementers consider cultural needs in a dynamic manner that does not ask patients to choose between meeting their cultural needs and receiving necessary health care during the postnatal period.

## 1. Background

### 1.1 Postnatal Care in Low and Middle Income Countries (LMICs)

Maternal mortality has fallen over the past several decades.^1^ However, maternal morbidity and mortality remain very high during the postpartum period, defined as the first six weeks following a birth. ^2^ Globally, most maternal deaths occur during the postpartum period.^3^ LMICs have higher rates of maternal mortality than high-income countries.^4^ In LMICs nearly half of maternal deaths occur within one day of delivery and one-fifth during days 2–7 following delivery.^5^ In Ethiopia, intrapartum and postpartum deaths account for the majority of maternal mortality.^6^ In Ethiopia, a high number of maternal deaths occur within 48 hours of birth.^7^ While maternal morbidity is difficult to calculate, rates are higher in LMICs than in high-income countries (HICs).^8^ To address maternal morbidity and mortality, women need access to high-quality postnatal care (PNC).

Postnatal care is the evidence-based intervention (EBI) that forms the basis of our implementation study. A systematic review of the literature states that home visits are effective in preventing maternal and neonatal deaths.^9^ Several randomized controlled trials have shown that home visits by community health workers are effective at reducing perinatal mortality in resource limited settings.^10–12^ Evidence from three randomized controlled trials indicated that these visits lowered perinatal mortality by 18%.^13^

The 2018 Ethiopian National Guidelines on postnatal care recommended that patients stay in the hospital or health center for at least 24 hours after giving birth.^7^ For healthy women, the World Health Organization recommends a minimum of four postnatal care (PNC) contacts, to be conducted within 24 hours after birth, between 48 and 72 hours after birth, between 7 and 14 days after birth, and during the sixth week after birth.^2^ PNC visits in Ethiopia are typically performed by female health extension workers (HEWs) at the patient’s home or at her local health post. ^14^ The Ethiopian Ministry of Health has provided guidelines that state women who deliver at home should receive three postnatal visits, while for institutional deliveries, two PNC visits should commence on day two (48–72 hour), as the first PNC visit will be given within the first 24 hours of postnatal care in the health institution of delivery. HEWs perform all home visits..^14^ These visits are performed with infant neonatal and well-baby visits. Recent data have shown that the incorporation of HEWs has had an insignificant effect in increasing PNC visits.^15^

However, according to a recent scoping review, in LMICs, postnatal care was the least utilized service across the continuum of maternal care, with the largest drop off in care coming after institutional births.^16^ In Ethiopia, only 34% of women had a postnatal exam two days after birth.^17^ Nearly one half of women who delivered their babies in hospitals or health centers did not receive their first PNC exam.^17^ Despite efforts to increase PNC^18^ and much quantitative data on facilitators and barriers to care^1919 – 21^, little is known about the role of cultural safety in accessing PNC in these settings.^22^

Cultural safety is a concept developed by Māori nurses in New Zealand that addresses the relationship between a patients’ cultural needs being met alongside positive health outcomes.^23^ Community and cultural considerations are at the core of cultural safety. For example, perinatal patients reported that kinship networks were critical to achieving cultural safety.^24^

The Ethiopian Ministry of Health set a goal of increasing the percentage of national adherence to 82% by 2025 and to increase the number of women who stay in the hospital or health center for 24 hours after giving birth by 25%.^6^ Much of this work will be done on the community level and will involve the influence of community and religious leaders.^3^

### 1.2 Barriers and Facilitators to PNC in Ethiopia

Barriers to PNC include being single versus being married, not being informed about PNC services, and experiencing overall health and not perceiving oneself to be in need of medical care.^25,26^ Facilitators for Ethiopian women receiving PNC include counseling from health providers, delivering at a health facility, having a Cesarean delivery, experiencing delivery complications, possessing an awareness of maternal health complications, outcome of birth (live versus stillbirth), and having a higher level of education.^21,25–28^ Higher socioeconomic status is also strongly associated with receiving one PNC visit; however no correlation exists between higher socioeconomic status and more PNC visits.^29^

Women throughout Ethiopia have also reported the influence of local religious leaders as significant as to whether they utilized health facilities.^30^ Many birthing women and health workers discuss how religious ceremonies which favored women giving birth at home or which are celebrated in the months leading up to birth (e.g. Muslim porridge ceremony) prevented many women from wanting to stay in maternity waiting facilities long enough to receive care.^31^ Moreover, beliefs about seeking religious support rather than health care support when health issues arose during pregnancy persist.^31^

The availability of and relationship between HEWs and the community are another mediator to women receiving PNC. Differences in expectations sometimes existed for HEW job description and performance.^30^ Many HEWs reported that their high workload prevented them from completing required duties.^30^ Moreover, while a referral system exists for patients, it does not always function well, due to communication breakdown at the health center and hospital level.^30^ This lack of communication was sometimes due to fractured relationships between HEWs and facility staff and because some women leave facilities before scheduling a PNC visit can take place.^18^ HEWs therefore do not always have access to information about the health of mother or the birth, especially for people who give birth at home, many of whom do not contact the health facility for fear of discrimination due to the location of their birth.^18^ Similarly, women’s communities sometimes hide births so that religious-cultural rituals can take place. Beyond this, logistical issues such as lack of telephone often makes such contacts between HEWs and postpartum mothers difficult.^18^ A study of HEW interactions during the postnatal period in rural parts of Northern Ethiopia found that health systems should develop better mechanisms for notifying HEWs of births and the need for visits.^19^ A study of the use of birth notification cards to communicate between health facilities and HEW in Southwestern Ethiopia showed an increase in PNC coverage.^32^

While HEWs expressed high motivation to make PNC visits, they reported that their supervisors did not support the visits.^18^ Many HEWs reported lack of interest from all levels of the health system in PNC, evidenced by lack of reporting systems for PNC visits, lack of supplies for PNC visits, lack of a clear job description, lack of transportation to reach families (especially those who live outside of the kebele), health structure politics, and a high workload.^18^ This lack of recognition and support for PNC reduces HEW motivation. ^33^

Current constructs of individual use of postnatal services cannot account for the societal and economic and sociopolitical forces deeply imbricated in all aspects of maternal-child care. This study employed abductive analysis using the Health Equity Implementation Framework^34^ (HEIF) to reexamine barriers to implementation in Ethiopian PNC.^30,31^ This approach adds complexity to perceptions of maternal decision-making. We previously found that patients exercise agency to protect their cultural safety during the postnatal period. ^22,35^We now examine the role of preservation of cultural safety in the utilization of postnatal care or lack thereof.

## 2. Materials and Methods

### 2.1 Study setting

Ethiopia has four levels of administrative divisions: regions, zones, woredas, and kebeles.^3^ Each *woreda* has a primary hospital and five health centers on average. A *kebele* is the smallest government health unit, which has one health post. The Ethiopian Ministry of Health assigns two HEWs to each *kebele* and its health post, which serves 5000 people.^30,36^,^31^

### 2.2 Study design and sampling

This study used data collected as part of an earlier study that evaluated a district-wide health systems quality improvement intervention in Ethiopia by the Institute for Healthcare Improvement (IHI) and the Ethiopian Ministry of Health using mixed-methods.^37^ Semi-structured, in-depth interviews were conducted between March and April 2018 and in April 2019. The study team and health leaders at each study site collaborated to purposefully select one hospital and one health center in each woreda that was representative of the catchment area’s diverse birthing experiences.

The sample included 63 women who had recently given birth in three Ethiopian regions: Southern Nations, Nationalities, and Peoples’ Region (SNNPR), Oromia, and Afar regarding their experiences receiving health care during recent deliveries. Participants gave verbal informed consent to participate in audio recorded interviews. Interviews were then conducted by Addis Ababa-based female research assistants (RAs), each of whom had graduate-level training in public health and two RAs in Afar, one of whom had graduate training and one of whom had undergraduate training. The semi-structured interview guide focused on participants’ most recent pregnancy and delivery. Open-ended questions were informed by the Donabedian Framework and included elicited information on staff interactions, services, and facility.^38^ We use pseudonyms throughout the text to refer to participants.

The interview process was iterative. Ras produced field notes that reflected their immediate reflections on interviews and regularly debriefed with the study team following each interview. These were used to refine the interview guide for future interviews to delve deeper into topics emerging in the data and achieve concept saturation.^39^ Interviews were transcribed verbatim in the language in which they were conducted. Interviews conducted in Amharic were translated to English for analysis. Interviews conducted in Afan Oromo, Afar, Dorze, Gamo, and Wolayetegna were first translated to Amharic before being translated to English.

The University of North Carolina at Chapel Hill’s Institutional Review Board and Ethiopian Public Health Association approved this research. Boards deemed the research program evaluation and thus the project was deemed exempt.

### 2.3 Data analysis

**Figure 1:**
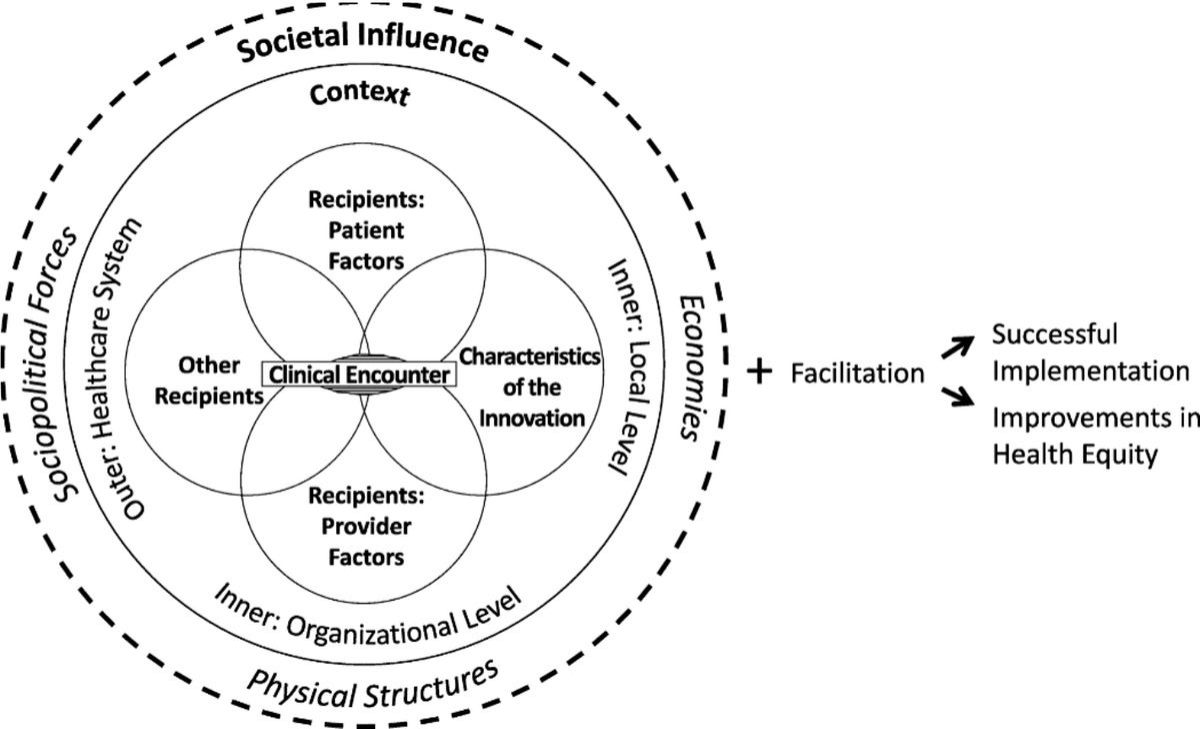
The Health Equity Implementation Framework

The Health Equity Implementation framework (HEIF) was developed as a combination of the Integrated-Promoting Action on Research Implementation in Health Services (i-PARIHS) framework^40^ and the Health Care Disparities Framework^41^ and “explains factors relevant to implementation and disparities in healthcare.”^34^ HEIF is represented in Fig. 1. We chose HEIF as our abductive analytic anchor because it proposes that investigation of societal influence should be integrated into analysis of all implementation factors, rather than being reserved primarily to the outer context.^34^ HEIF proposed three domains to address health equity that could be added to any determinant framework.^42^

HEIF centers the clinical encounter is the point at which the innovation is delivered. The clinical encounter is characterized as an interaction between recipients and the innovation. The inner context is comprised of both the local and organizational levels, which correspond to the clinic and hospital or network, respectively. The outer level is defined as the healthcare system. Finally, the model considers societal influence on innovations through sociopolitical forces, physical structures, and economies.

We took an abductive approach to analyzing interview data, using the codebook developed by the study team at the time of the first study and HEIF.^43^ We chose this approach because we used the HEIF to understand our inductive and deductive interview data and because our data added to the framework.^43^ The codebook was developed through regular debriefs and meetings to discuss interview, transcripts, and code production. It included metacodes reflecting context, healthcare experience, phase of care, relationships, satisfaction, and equity and disparities and codes within each of these categories. Transcripts were originally coded using ATLAS.ti and NVivo.^44^ The team double coded half of the transcripts, met to resolve coding discrepancies, and finalized code definitions when there were no longer disagreements between team members regarding codes. The remaining transcripts were single coded. We applied deductive codes to participants’ narration of their experiences using the Donabedian quality care framework.^38,45^ Inductive codes were developed through in-vivo coding, idioms and euphemisms, and repetition-based theme analysis. Structural codes such as age group of participant, ethnicity, and birth facility type were also applied.

In our abductive analysis, the team first returned to portions of the data that were coded for postnatal care. This code described mothers’ experiences receiving or not receiving healthcare services after delivery at health facility, home, or community setting. We then used NVivo to identify overlap between the structural postnatal care code and deductive and inductive codes to more deeply understand why implementation of the Ethiopian postnatal care program has not been successful. Overlapping codes were then grouped using an abductive approach using the domains of the HEIF and arranged as facilitators and barriers to accessing the clinical intervention of PNC.

The following is an example of our analytical process: where the codes postnatal care overlapped with discrimination/prejudice code and where postnatal care overlapped with structural barriers, we mapped these codes onto “sociopolitical forces” in the Health Equity Implementation Framework using an abductive approach.

### 2.4 Reflexivity Statement

Our research team included Ethiopian and American academics with backgrounds in history of medicine, public health, and anthropology, Ethiopian medical providers and public health professionals, and Ethiopian public health researchers. While all of us were outsiders with distance to our participants, our varying backgrounds generated different approaches to our data and this project. The research assistants who collected our data had more in common with our participants in terms of life experiences and therefore were able to understand some cultural nuances. The authors who led the analysis were two white American academics who have experience giving birth and navigating culturally challenging health environments. We remained close to our data and consulted our in-country research team when we needed clarification. We consider our project a collaborative contribution to discussions of postnatal care use, individual agency, and implementation science, rather than an objective statement of fact.

## 3. Results

We anchor our findings around utilization of PNC among Ethiopian while considering their preservation of cultural and physical safety as an expression of agency and as critical to their care. We will describe the findings by domain.

### 3.1. Clinical Encounter: Giving control to God

#### 3.1.1 Recipients: patient factors: Culturally safe foundations

Patient were imbricated in culturally safe systems of traditions and faith that they trusted more than the care of the health system. Their prioritization of these systems over PNC within the formal health system was often viewed as a barrier to care. We argue instead that the choice to not leave home after birth and an overall perception that God’s will was most critical to one’s health and that of her baby, were an expression of agency in order to maintain cultural safety. Table 1 shows the PNC codes as they correlate with HEIF domains and subdomains.

Women expressed their care preferences within a social structure that they saw as critical to their well-being and a belief structure that they saw as having ultimate control over their lives. While this did not conclusively preclude them seeking care for medical appointments, they expressed a preference for staying in their homes following birth. Aleke, a Christian woman from SNNPR, reflected on the custom of not leaving one’s house for several weeks following birth. When asked when she left her bed to resume daily life following her baby’s birth, she responded: “I am not out yet; you have to wait 80 days for a baby girl and 40 days for a boy ….” (26 years, 2 live births, Orthodox, SNNPR). Although she had an appointment scheduled for the next day, she affirmed that she did not want to go. Chaltu, a Christian woman from Oromia elaborated on this custom, called Christina: “After delivery a woman is not allowed until she go to church and pray. She is not allowed to enter to the kitchen until that. There is a ceremony called ‘Christina’ which means simply giving baby to God. It is held after 80 days, until then the delivered woman shouldn’t work outside. This is what religiously not allowed” (35 years, 3 live births, Orthodox, Oromia). While illness would be considered an exception to this custom, Chaltu said that, thanks to God, she had remained well and therefore continued to remain in her house. After Christina had taken place, she affirmed that she “could go everywhere,” as well as return to her domestic duties.

Regardless of religious customs that interfered with the logistics of seeking care, many women reported that they believed the outcome of the pregnancy and birth processes were in God’s hands and did not perceive themselves as playing a decisive role in the outcomes of their pregnancies and births. Ebise explained that part of the welcoming ceremony for her and her baby upon returning from the health center was thanking God for the safe delivery of her baby. The welcome ceremonies underscored the connection between community support and cultural custom. She explained, “According to our culture coffee ceremony was prepared; Injera was baked; everything was ready, people brought coffee, they hugged and kissed me saying ‘congratulations!’ Thanks to the God for separating you from your offspring safely.” (40 years, 4 live births, Orthodox, Oromia). The cultural customs for the postpartum period were deeply imbricated in a religious context of God’s will regarding the wellbeing of mothers and babies in the weeks following birth.

While the women in our study delivered in health centers and hospitals and many said they would seek future medical care when necessary, most said they considered their health to ultimately be in God’s hands. When asked about her expectations about future pregnancies, Jemila answered, “that God is the one who give child” (18 years, 1 live birth, Muslim, Afar). Another participant, Kimia, explained that medical intervention could only go so far: “What will they give me only the blood they give me and the drugs they do and lastly its God who help me to be healthy.” When asked about the role of health workers, Kimia continued, “Yes it was God’s will but they help me from their side and nothing left from the things they do if God doesn’t will I would have been sick and not healthy….. you see… so God help me with it” (20 years, 1 live birth, Muslim, Afar). Kimia’s responses reflect how participants described an external locus of control in which their actions played only a small part in the safety of them and their babies during this period. Kimia’s experience highlights how women often did not seek PNC because they considered themselves to be well. As Kimia explained, because she did not perceive symptoms of unwellness during the postnatal period, she deemed herself well and that God was maintaining her health.

In these three Ethiopian regions, many women appreciated the benefits of health care and health providers but perceived their benefits to be an extension of God’s will. Community support provided assistance that was consistent with these religious beliefs. When women did leave their homes to seek care, it was usually for a clearly adverse health event, such as excessive bleeding.

#### 3.1.2 Recipients: Provider factors: Pursuing useful care

Women were more inclined to receive care when the HEW visited their homes, because it enabled them to maintain their cultural safety and satisfy care recommendations for them and their babies at the same time. When asked whether she made an appointment for PNC at the hospital Dalbore explained “No, I don’t have an appointment …The health care provider comes home and immunizes the baby and check up on my health status” (30, 3 live births, Protestant, SNNPR).

Moreover, when they built trust with their HEW, women described following through with care, as well as developing trust in the health system generally. When asked about her relationship between her HEW and mothers in her community, Dalgite responded that the HEW’s consistency had led to a positive relationship in which the mothers received support, ongoing education, and healthcare such as vaccines. She described how her HEW provided her baby vaccines, gave her breastfeeding support and health information regarding her pregnancy, as well as her confidence in the HEW. She also expressed confidence in the education that the HEW provided: “I know the education is a good thing for my baby” (30 years, 3 live births, Protestant, SNNPR). When HEW care was consistent and deemed useful by participants, they were receptive to receiving it.

#### 3.1.3 Characteristics of the Intervention: Importance of regular contact and home contact

Some women who received PNC reported that it was convenient to do so when the HEW came to their homes and when the performed care for their babies at the same time. The model of dyadic care for mother and baby, as well as an overall confidence in vaccination that participants expressed, made receiving care more culturally safe for them. When HEWs were able to visit homes, women could access care, as well as care for their newborns while maintaining cultural safety. Moreover, regular contact with HEWs built trust between them and the community and made women more inclined and more able to access such care when it was available to them. Dalgite explained of her HEW, “She comes every day whether she has a vaccine appointment or not.” While women did not commonly report that HEWs visited them every day, several who utilized postnatal care received it through regular visits in their homes.

### 3.2 Inner context: Lack of organizational and system communication

Our findings show that many women did not attend a PNC visit because they were not told such a visit was necessary. Overwhelmingly, our findings suggest participants were not made aware of the necessity of postnatal care.

#### 3.2.1 Inner context: Local level: Lack of communication

Women’s agency was limited by not knowing about the importance of or recommendation for PNC appointments due to lack of communication between the health facility and themselves, as well as the health facility and the HEW. Table 2 shows the PNC codes as they correlate with HEIF domains and subdomains. Women across all three regions reported that no one told them they needed to schedule a PNC appointment and or asked them to schedule one. Maryam explained, “They didn’t tell me and I don’t know; They didn’t say anything about visit experience or practice after delivery …” (20 years, 4 live births, Afar, Muslim). Others participants said they were told to come back only if they experienced an obvious health issue, such as bleeding. Some, such as Kimia, sought care for health conditions and said they only went because of these issues (24 years, 4 live births, Afar, Muslim). Table 3 depicts responses from each respondent who reported that she was not told about the need for PNC.

The above findings underscore that women exercised their agency in accessing care when they were adequately communicated with and regarded that care as necessary.

#### 3.3.2 Inner context: organizational level: Insufficient care networks in system

Some participants reported that the clinical and hospital network did not meet women’s needs for care for their babies. Some women reported seeking PNC would require them to leave their babies at home, something they did not want to do. One participant declined to leave her home despite experiencing an obvious health issue, because she could not take her baby. She explained, “I ask them what should be done if there is bleeding? And they told me that they will give medication and told me to come back after 6 weeks. But I did not go…was thinking about how could I go holding my baby, So I did not go.” These findings on the organizational level build on those from the local level and explicitly highlight limitations of understanding use of PNC within the Ethiopian health system as a choice made by individuals.

#### 3.2.3 Outer healthcare system: Self-protection after mistreatment:

Some women’s previous healthcare experiences influenced whether they chose to seek additional care. Maryam expressed that she had lost trust in the health center after the inadequate care she received during her second pregnancy. “In previous times they check us and see if something is wrong. They tell us to come to the center if we feel dizziness. Even I check myself without my appointment. They take every examination if there is symptoms they treat us immediately. This recent one I don’t know what they did for me. (20 years, 4 live births, Afar, Muslim). Her dissatisfaction extended to care her baby had received. Referring to a health visit they had attended together, she explained: “They didn’t watch him properly,” she explained. “just give us syrup. I gave 30 birr for that come back to my house.” She went on to clarify that she wanted to seek care for herself, but would not do it in the health center where she had been mistreated. “P: I like to be treated by health care,” she said. “I don’t think I will stop myself from going there and things happen with GOD’s will. But I don’t go to [the health center]for delivery.” She explained that in addition to lack of care, she had been mistreated during birth by being attended to by a male provider, which went against her religious beliefs and because staff did not know how to deliver her baby due to her scar from female genital cutting. Although she was experiencing an illness during the interview, she was not motivated to visit the health center until after her 45 days: “What will they do if we go to them?”

For Maryam seeking PNC was not worth the physical or financial risk to her and her baby. Her story emphasizes how women made active choices to protect themselves in situations in which the health system did not provide adequate or safe care for themselves and their babies. Their experiences within the health system, their desire for self-preservation, and the difficulties of navigating the insecurities of the health system strongly influenced women’s willingness and ability to continue care within the health system and influenced their choices around receiving such care.

#### 3.3.3 Outer context: healthcare system (formal and informal)

Women negotiated faulty health systems and logistical issues to receiving care and often perceived themselves to be best supported in their home communities, which facilitated practical care, as well as their cultural requirements. Table 4 shows the PNC codes as they correlate with HEIF domains and subdomains.

#### 3.3.1 Sociopolitical forces: Logistical barriers to care

Women took more action to receive health care that they perceived to be important for themselves and their children. Vaccine recommendations for both infants and mothers were an effective motivator for women following up on PNC recommendations. Lechame described how her social network enabled her to stay home with her baby for three months after her birth, but vaccination prompted her to leave her compound. She explained, “They stay for 4 months or so… for me it has been 3 months and I have not left the compound yet. I: What about for vaccinations and your follow-up P: For that I will go, I did go…I went for follow-up on the 5^th^ day. For vaccination I went one day with the baby” (28 years, 2 live births, Protestant, SNNPR). This was a concrete health intervention they understood they needed, versus a recommendation for PNC they perhaps did not think they needed because of their relative health.

Family members and neighbors deeply influenced women’s daily lives in that many were cared for in their homes and did not seek help elsewhere and because of deeply embedded social traditions around birth. These social networks functioned as an informal care network that in some ways replaced the formal health system.

#### 3.3.2 Finances

Several participants described complex risk weighing around the value of health visits versus the resources these visits would cost them. Halima described accessing health care as contingent on her finances, which were especially stressed after she lost some of her money on the way to a hospital in Addis Ababa to seek care for her baby: “when we enter [the hospital] the doctors ask me if I have money… I told them I have enough money and he can see my account from the bag .he said yes you have enough. Thanks to God, if I couldn’t have the account book I don’t know what will happen because I lost the 30 thousand birr (26 years, 5 live births, Muslim, Afar). Others, like Rahima, said even when experiencing an obvious health issue she had been warned about, they did not go to the health center because of lack of financial means. When asked why she didn’t take her baby to the health center, she responded, “I was out of money and my focus was on my baby… transportation is very problematic to go to Berta and you have to walk far distance to reach the health centre.” (20 years, 1 live birth, Protestant, Afar). However, for women to whom PNC valued a visit, they worked hard to overcome barriers to receiving it.

## 4. Discussion

Our study addressed the set of goals developed by the Ethiopian Ministry of Health to increase demand for PNC by 2025 (RH Strategic Plan).^7 30,31^ We employed an abductive analysis using our inductive-deductive codebook and the Health Equity Implementation Framework to identify novel understandings of end users interfacing with PNC in Ethiopia.^46,47^ Our contributions converge around how understanding that patients seek to maintain their cultural safety reframes many so-called culture-based “barriers” as a continuous expression of agency.

By presenting patients as acting to preserve their cultural safety, our paper adds important complexity to implementation research literature on health equity.^42,48,49^ The HEIF draws focus to community and cultural factors that influence individual choices and adherence to health protocols.^34,41^ With its requirement that patients must determine whether cultural safety is met, it responds to the emerging standard of engaging end users in health equity research,^50^ which early evidence suggests can be useful in increasing health behaviors and improving outcomes.^51^ Approaching end users as maintaining their personal safety also speaks to the goals of implementation science to tailor interventions to a specific community,^52,53^ reflexivity on behalf of health systems and scholars in implementation science,^54^ and more standardized methods for increasing end user engagement,^55,56^ while acknowledging that end users are imbricated within complex contexts.

**Figure 2:**
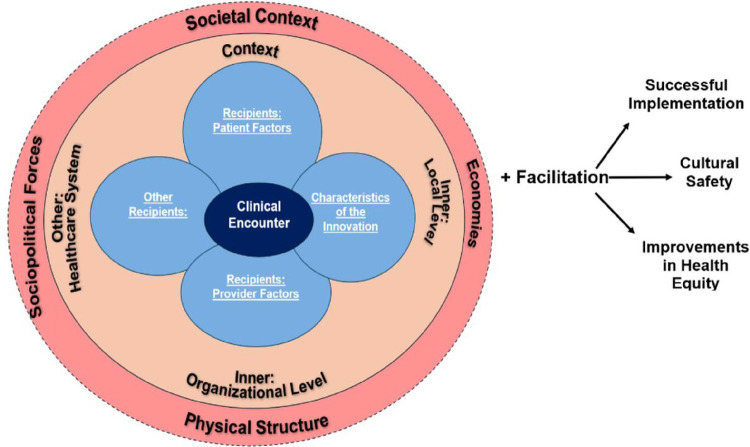
Health Equity Implementation Framework with proposed cultural safety addition

We propose the addition of a process outcome of cultural safety to HEIF to highlight its role in increasing service utilization and thereby, increasing health outcomes, as portrayed in Figure 2. Studies have already noted that cultural safety leads to better health outcomes in LMICs.^57–60^ This study joins emerging scholarship that focuses on quality of care in the wake of the World Health Organization’s call for an emphasis on this theme.^2,35,45^ When women perceived care as important, they took steps to access it. For example, many women understood vaccination as an important intervention for both them and their babies and actively pursued this healthcare even when it required temporarily breaking with their customs. Implementation strategies must therefore account for how patients frame trust within health systems, which influences how women make their decisions about their health when proposing educational interventions.

Maternal health researchers cite postpartum care as a top priority for implementation research.^61^ However, maternal-child health interventions remain underrepresented in implementation research and most literature has focused on facilitators and barriers to care, rather than applying theories, models, and frameworks.^62^ Furthermore, while some of these theories, models, and frameworks examine the aspects of context that contribute to individual adherence to health interventions, the role of culture and societal influence in people’s adherence remains largely underexamined.^63,64^ Of the few implementation science interventions in maternal-child health papers, several have cited determinant frameworks, such as the Consolidated Framework for Implementation Research (CFIR). The CFIR 2.0 Framework includes a Societal Pressure domain that is divided between Roles and Characteristics.^64^ HEIF allows for further breaking down of the individual domain with focus on patients, providers, other recipients, and the clinical encounter and places these encounters between individuals at the center of analysis.^34^ Our inclusion of cultural safety as a process outcome within HEIF extends this literature by addressing individual barriers and facilitators to receiving care as imbricated in a larger structure, rather than as separate domains.

Our paper also joins recent implementation research in LMICs that has pointed to context-specific needs in the education and training of healthcare workers^65,66^ and the need for respectful maternity care in LMICs. ^67,68^ Satisfaction with care is increasingly recognized as a necessity for quality care. ^52^ The inclusion of cultural safety provides the means for achieving cultural respect across contexts with its principles of ongoing provider reflexivity,^23,69^ rather than a prescribed list of requirements for respectful care. ^24,70^

Cultural safety also provides a way of thinking through the “black box” of matching implementation strategies onto barriers to care.^71^ A review of perinatal participants’ understanding of culturally safe interventions highlights birthing in community, acknowledgement of difference, and respect for culturally situated knowledge.^24^ This emerging knowledge allows of cultural safety’s use in health encounters allows us to map implementation strategies onto HEIF more clearly.^72^ Create learning collaborative^72^ addresses need for acknowledging difference and respecting end user’s knowledge.^24^ Involving patients and family members^72^ addresses need to experience health care in community. Obtaining and using user and family feedback^72^ explicitly references cultural safety’s requirement that patients decide whether cultural safety has been achieved^23^ Several validated tools for evaluating and implementing culturally safe care exist that have been tested in HICs. Future studies could test strategies that have been successful in HICs such as training providers and administrators in skills such as active listening^73^ in LMICs. Additionally, future studies could test assessment of cultural safety^74^ and evaluation of cultural safety programs in LMICs.^75–77^ This will add to calls for clear clinical definitions of cultural safety so that interventions can be more culturally safe.^78^

Utilizing these tools at every level of the health system will takes emphasis off patient, who, as non-experts, cannot be expected to state their health needs thoroughly. This systems-level approach also takes emphasis off hyper-burdened providers and the clinical encounter:^79^ Training of clinicians in cultural competency has had so far limited or poor effects^80–82^ Rather, an approach to cultural safety must be integrated into the whole health system in order to effectively engage end users in their many contexts.

### 4.1 Strengths and Limitations

Our study has limitations. First, because we only sampled from three Ethiopian regions, our study may not be transferable to women in all regions of Ethiopia. However, our results may be transferrable to other LMICs. Linguistic nuances could have been lost in the process of doubly transcribing interview audio into English. We asked women to describe their prenatal, birth, and postnatal experiences, many of which included intense [things]; this could have led to women feeling uncomfortable sharing, despite rapport building and establishing consent on behalf of the interviewers.

## 5. Conclusions

Our analysis provided insight as to how social, cultural, and structural factors overlap influence women’s decisions to seek care during the postpartum period. Our findings indicate that women’s agency guides their decision-making based on how they perceive their well-being to maintain their commitment to cultural and religious practices, obtain support within their community, and to limit potentially adverse healthcare experiences. Utilization of a health equity implementation framework ensures that these multiple and overlapping factors address the importance of social place and agency when mothers make decisions about postnatal care.

## Figures and Tables

**Table 1.** Matrix of barriers: themes relating to the HEIF clinical encounter

HEIF Domain	HEIF Subdomain	PNC Codes	Health action or factor	Facilitated or impeded PNC
1. Clinical Encounter	1a. Recipients: patient factors	Healthcare expectations	Was informed	Facilitated
			Was not informed	Impeded
		Feelings	Happiness of family members for using health care	Facilitated
		Traditions & customs	Staying home 40 days after birth	Impeded
			"Everything in God's hands"	Impeded
			Not feeling bound to traditions and customs	Facilitated
			Postpartum health issue	Facilitated
			Baby health issues	Facilitated
	1b. Other recipients: family members, other participants in the EBI, birth support person	Family members	Family supporting facility use	Facilitated
	1c. Recipients: Provider factors:	Trust	Trusting HEW	Facilitated
	1d. Characteristics of the Intervention		Dyadic care; HEW visiting home	Facilitated

**Table 2.** Matrix of barriers: themes relating to HEIF inner context

HEIF Domain	HEIF Subdomain	PNC Codes	Health action or factor	Facilitated or impeded PNC
2. Context factors Inner context: local level	2a. Inner organizational level	Facility delivery	Received inadequate care	Impeded
			Did not want to leave baby	Impeded
	2b. Inner local level	Postnatal care	Never told to return	Impeded
	2c. Outer healthcare system		Consistent HEW visits	Facilitated

**Table 3:** Participant responses to being asked if they were told about importance of PNC[1]

IDI	Response to being asked about PNC
IDI 02	"I donť know that . do we need to go even if my baby is doing well."
IDI 04	"They [only] told me that there is vaccination for me and my baby after 45 days."
IDI 06	" After delivery they didnť tell me to come back."
IDI 07	Was told to visit, "if your children are sick and something wrong with them... and after two days of delivery if your blood didnť stop."
IDI 08	" No, no one give me appointment [after delivery]"
IDI 10	" nobody told me something like that" [to come after delivery]
IDI 12	" Interviewer: no one counsel or appoint you after you gave birth to come back for postnatal care?: P: "Yes"
IDI 16	"No one give me appointment. I went to my house after one day."
IDI 17	"What kind of follow - up?... we go to the center to vaccinate the baby."
IDI 18	Interviewer: "no one gave you information or counselling to come after giving birth for post natal care follow up? Participant: "yes"
IDI 27:36	" They didnť say anything, they just [discharged me]."
IDI 27:35	" I didnť learn about that after delivery."
IDI 39:69	"I didnť have an appointment."
IDI 27:98	"They didnť say anything, they just told me to go out."
IDI 39: 77	"They did not appointed me."
IDI 42:#	"I did not get an appointment."

**Table 4:** Matrix of barriers: themes relating to HEIF outer context

HEIF Domain	HEIF Subdomain	PNC Implementation Theme	Health action or factor	Facilitated or impeded PNC
1. Societal context	3a. Sociopolitical forces	Vaccines	Infant vaccines	Facilitated
			Maternal vaccines	Facilitated
	3b. Economies	Finances	Not having enough money for appointment	Impeded

## Data Availability

Data is available upon reasonable request directed to author Professor Ashley Hagaman (ashley.hagaman@yale.edu).

## Abbreviations

PNC: postnatal care
LMIC: low-and-middle-income countries
HIC: high-income countries
EBI: evidence-based intervention
HEIF: Health Equity Implementation Framework
HEP: health extension program
HEW: health extension workers
SNNPR: Southern Nations, Nationalities, and Peoples Region
CFIR: Consolidated Framework for Implementation Research
